# Zoster Sine Herpete: Confirmatory Diagnosis Using Varicella-Zoster Virus DNA Polymerase Chain Reaction Analysis of Intact Skin Scrapings

**DOI:** 10.7759/cureus.41965

**Published:** 2023-07-16

**Authors:** Sean Ho Yoon, Renuka Shetty-Das

**Affiliations:** 1 Internal Medicine, NewYork-Presbyterian Queens, New York, USA

**Keywords:** skin test, zoster sine herpete radicular pain, post-herpetic neuralgia, shingles complications, varicella-zoster virus

## Abstract

Zoster, commonly known as shingles, is a syndrome caused by the reactivation of the varicella-zoster virus (VZV) that results in characteristic vesicular eruptions in a unilateral dermatomal distribution. Timely diagnosis of zoster sine herpete (ZSH), a variant of shingles without the typical vesicular rash, can be challenging due to the absence of visual cues and limited application of traditional diagnostic methods. Recent case reports have demonstrated the utility of VZV DNA polymerase chain reaction (PCR) analysis of involved skin exudates for confirming ZSH. This report presents a case of acute ZSH involving the left trigeminal nerve, where the diagnosis was confirmed using VZV DNA PCR analysis of skin scrapings. The patient initially presented with radicular pain and was treated for presumed shingles, but persistent neuropathic pain persisted. The confirmatory VZV DNA PCR analysis of the involved skin scraping sample aided in establishing the diagnosis of ZSH. The case highlights the potential of using intact skin scrapings for VZV DNA PCR in confirming ZSH. Prompt initiation of antiviral therapy is crucial for minimizing the duration and severity of radicular pain in ZSH cases. Larger studies are needed to further evaluate the utility of VZV DNA PCR analysis for accurately diagnosing ZSH.

## Introduction

Zoster, or shingles, is the reactivation of the varicella-zoster virus (VZV) that manifests as clusters of characteristic vesicular eruptions distributed unilaterally in a dermatomal pattern. In the United States, approximately 1 million cases of herpes zoster occur annually as of 2015, approaching a 30% individual lifetime risk [1]. The rash is preceded by severe burning pain, dysesthesia, or hyperesthesia in the affected area. Following an initial VZV infection in childhood, the pathogen enters a latent period in a posterior dorsal root or Gasser ganglion, which can be reactivated later in life [2]. Shingles is primarily diagnosed based on clinical presentation, as it displays a visually apparent and clear temporal regional pattern of the rash. However, timely diagnosis can be challenging in cases of zoster sine herpete (ZSH), an uncommon variant of shingles, due to the absence of the characteristic vesicular eruption required for clinical judgment and the limited applicability of alternative traditional virological or serological methods [3]. Recently, a few case reports have shown that ZSH can be confirmed through VZV DNA polymerase chain reaction (PCR) analysis of involved skin exudates [4-6]. In this article, we present a patient case of acute ZSH involving the left first and second divisions of the trigeminal nerve, where the diagnosis was confirmed by using skin scrapings from the affected area for VZV DNA PCR.

## Case presentation

The patient was a 74-year-old Hispanic female who presented to our clinic with a complaint of sudden onset constant burning, and hyperesthesia in her left-sided upper facial area, which involved the left scalp, frontal, ophthalmic, and maxillary regions for a few weeks. Prior to the clinic encounter, the patient visited the hospital emergency department one week after the initial onset of symptoms and was prescribed acetaminophen, oxycodone, and valacyclovir for seven days for presumed shingles. Despite these treatments, she experienced persistent neuropathic pain without visible skin findings. The patient had also been seen by a neurologist for a known chronic non-specific neuralgia-type headache and had briefly taken carbamazepine without symptomatic relief, which was then switched to gabapentin 300 mg three times daily. On physical examination, facial skin findings were unremarkable, with an absence of vesicles or erythematous patchy scales. Palpation on the left upper facial area elicited sharp tenderness and a burning sensation. Laboratory tests showed no leukocytosis, a sedimentation rate of 62 mm, and a C-reactive protein level of 0.08 mg/dL. 

A follow-up neurology visit was made with concerns of worsening neuralgia-type headaches or paroxysmal hemicrania. However, the mentioned diagnoses were deemed unlikely based on the lack of clinical improvement despite the previously described multimodal medical regimen. Upon reassessment, clinical suspicion for presumed post-herpetic neuralgia was raised, and the dosage of gabapentin was subsequently increased to 800 mg three times daily for optimal symptom control. 

To definitively diagnose VZV involvement, a skin scraping sample was taken from the affected area for VZV DNA PCR, which yielded a positive result. The serum varicella zoster IgM antibody test, however, was negative. With the confirmed diagnosis of ZSH based on the skin sample, the patient was prescribed 3% lidocaine topical cream as needed, in addition to continuing with gabapentin. Over a few weeks, her radicular pain gradually improved, as observed during repeat cranial nerve examinations at subsequent close follow-up visits. The patient was also referred to an ophthalmologist, who confirmed the absence of herpes zoster ophthalmicus.

## Discussion

Zoster sine herpete is a condition characterized by radicular pain along a dermatomal distribution without the presence of an erythematous or vesicular rash, which is typical of shingles [3]. The diagnosis of shingles is often made clinically, based on the visually apparent and clear temporal-regional pattern of the rash. In cases where the clinical assessment is inconclusive, traditional methods such as VZV DNA PCR, Tzanck smear using herpetic vesicle specimens, or the detection of serological markers with immunoglobulins like serum IgM and IgA antibodies can be employed to confirm the diagnosis. However, ZSH lacks herpetic vesicles, and the serological response specific to IgM and IgA has been shown to be present in only approximately 60% of cases [7]. The limited applicability of traditional virological and serological methods makes the accurate and timely diagnosis of VZV infection challenging, especially when the patient has an unrelated existing chronic neurological condition, as in our case. Thus, the detection of VZV reactivation in ZSH requires not only a detailed history and physical examination but also the analysis of involved skin samples for confirmatory VZV DNA PCR.

Recent case reports have demonstrated the utility of VZV DNA PCR analysis of involved skin exudates in diagnosing ZSH. These cases involved vesicles or open skin lesions from vesicular eruptions that were subjected to PCR analysis, confirming the diagnosis of herpes zoster [4-6]. However, our case demonstrates that intact skin samples scraped from the affected area without any exudates or open vesicles can also yield the same outcome. In theory, since ZSH is a clinical variant of shingles, it consists of three stages based on "herpetic lesions": preherpetic neuralgia, acute zoster pain, and postherpetic neuralgia [8]. Therefore, we recommend initiating antiviral therapy against VZV as soon as clinical suspicion of ZSH arises, without waiting for confirmatory skin testing. Initiating treatment during the preherpetic stage, which typically occurs within the first 4-5 days, can minimize the duration and severity of lasting symptoms [9]. We suspect that the prolonged dermatomal neuralgia refractory to treatment in our patient could be attributed to a delayed intervention with antiviral therapy beyond the preherpetic stage. Nevertheless, larger retrospective case studies need to be conducted to further investigate the utility of VZV DNA PCR analysis of intact skin samples or exudates in confirming the diagnosis of ZSH.

## Conclusions

Our patient presented with isolated radicular pain along a dermatomal distribution of the left trigeminal nerve without the characteristic herpetic rash. The patient received early antiviral therapy for a presumed shingles infection, and later the diagnosis of ZSH was confirmed when VZV DNA PCR of the involved skin scraping sample yielded a positive result. Our case illustrates that scraping intact skin samples from the affected area, even without any exudates or open vesicles, can be used to confirm ZSH. Although the diagnosis of ZSH requires confirmatory testing, antiviral therapy against VZV should be initiated as soon as clinical suspicion for ZSH arises to minimize the duration and severity of radicular pain.
